# Synthesis of (R)-(6-Methoxyquinolin-4-yl)[(1S,2S,4S,5R)-5-vinylquinuclidin-2-yl]methanol Tetraphenylborate Ion-Pair Complex: Characterization, Antimicrobial, and Computational Study

**DOI:** 10.3390/molecules28196974

**Published:** 2023-10-08

**Authors:** Tarek A. Yousef, Haitham Alrabiah, Mohamed H. Al-Agamy, Rashad Al-Salahi, Essam A. Ali, Gamal A. E. Mostafa

**Affiliations:** 1 Chemistry Department, College of Science, Imam Mohammad Ibn Saud Islamic University (IMSIU), Riyadh 11623, Saudi Arabia; 2 Department of Pharmaceutical Chemistry, College of Pharmacy, King Saud University, P.O. Box 2457, Riyadh 11451, Saudi Arabiaralsalahi@ksu.edu.sa (R.A.-S.);; 3 Department of Pharmaceutics, College of Pharmacy, King Saud University, P.O. Box 2457, Riyadh 11451, Saudi Arabia.

**Keywords:** quinine, tetraphenyl borate, ion-pair complex, characterization, antibacterial activity, computational study

## Abstract

The (R)-(6-Methoxyquinolin-4-yl)[(1S,2S,4S,5R)-5-vinylquinuclidin-2-yl]methanol (quinine)-tetraphenylborate complex was synthesized by reacting sodium tetraphenyl borate with quinine in deionized water at room temperature through an ion-pair reaction (green chemistry) at room temperature. The solid complex was characterized by several physicochemical methods. The formation of ion-pair complex between bio-active molecules and/or organic molecules is crucial to comprehending the relationships between bioactive molecules and receptor interactions. The complex under study was examined for antimicrobial activity. All theoretical calculations were carried out in vacuum and water using the B3LYP level 6–311G(d,p) levels of theory. The theoretical computation allowed for the prediction and visualization of ionic interactions, which explained the complex’s stability. The results of energy optimization showed that the Q-TPB complex is stable with a negative complexation energy. The obtained geometries showed that the boron (B^−^) and nitrogen (N^+^) in piperidine of the two molecules tetraphenylborate and quinine are close to each other, which makes it possible for ions to interact. The modest energy gap between HOMO and LUMO showed that the compound was stable. The computation of the electron transitions of the two models by density functional theory (TD-DFT) in the solvent at the theoretical level B3LYP/6–311G(d,p) allowed for the detection of three UV/visible absorption bands for both models and the discovery of a charge transfer between the host and the guest. The UV absorption, infrared, and H NMR are comparable with the experimental part.

## 1. Introduction

Quinine is a cinchona alkaloid that contains carboline. Its chemical name is (R)-(6-Methoxyquinolin-4-yl)[(1S,2S,4S,5R)-5-vinylquinuclidin-2-yl]methanol. This naturally occurring alkaloid is derived from the bark of the Cinchona tree, which grows in India, South America, and Indonesia. Quinine was the first effective drug for treating malaria. Moreover, quinine and its derivatives have been extensively studied for their potential as antibacterial, antiviral, and antiarrhythmic medications. Moreover, numerous cancer cell lines have confirmed their cytotoxic qualities. Many attempts have been made to produce analogs with improved biological efficacy. For instance, quinine dimerization using ester bonds or quinine modification using copper-catalyzed azide-alkyne cycloaddition (CuAAC) produced compounds that were more hazardous to cancer cells and Plasmodium falciparum. Recent research has shown that squaramides and quinine-based thioureas, which function as hydrogen-bond donors, are remarkably effective as Cl-ion transporters. Through the activation of a caspase-dependent mechanism, these chemicals preferentially induce apoptosis in cancer cells [1]. Since the discovery of quinine’s anti-malarial effects, it has been used in medicine to treat this parasitic disease for more than three centuries [2,3].

Electroactive materials such as quinine-tetraphenyl borate [4,5] or a tetraphenylborate derivative [6,7] have been used to make quinine sensors. Non-covalent chemistry has introduced a number of interesting artificial receptors and developed host–guest chemistry, or supramolecular chemistry, over the last two decades. These multifunctional receptors have been particularly interested in sensing and recognizing various neutral chemicals and/or ionic entities (e.g., anions, and cations) [8,9,10] of specific interest.

The main objective of supramolecular chemistry is to achieve mastery of non-covalent intermolecular bonding. The cohesion of supramolecular assemblies made up of different entities is ensured by non-covalent bonds such as metal–ligand coordination bonds, hydrogen bonds, π-π interactions, hydrophobic, ionic interactions, and van der Waals forces. All these interactions have lower breaking energies than covalent bonds [11]. The stability of these molecular systems relies on the establishment of low-energy non-covalent interactions, which depend on the geometry and nature of the chemical groups assembled [11,12]. These intermolecular interactions form the basis of highly specific processes and play a fundamental role in various fields, with, for example, the attachment of a substrate to a protein receptor, enzymatic reactions, immunological antigen-antibody association, and all the other aspects of cellular communication [13]. Instance, supramolecular chemistry has resulted in the formation of a large number of fundamental compounds. These molecules were being researched for their potential uses in a variety of fields, including those dealing with biology [14,15], the environment [16,17], and pharmaceuticals [18,19,20]. 

In recent years, scientists have become interested in ion-pair receptors [21,22] that have separate binding sites for both cations, anions, and neutral substances. This is because ion-pair receptors are one of the many types of monotopic receptors. Ion-pair receptors are important in different application for example ion-pair recognition behavior [23], photoswitchable [24,25], and extraction and transport features [26,27,28]. We aim to synthesize, characterize, and investigate the quinine-tetraphenyl borate ion-associate complex because of the significance of the ion-pairs complex systems (as receptors, recognition behavior, photoswitchable, and extraction and transport capabilities). This is because of the significance of the ion-pair complex systems.

This study focuses at the experimental and theoretical studies of quinine-tetraphenyl borate ion-pair creation as an ionic liquid in water under ambient conditions as a green way to make an ion-associate or ion-pair complex. The quinine-tetraphenyl borate ion-pair complex is the focus of this study, which aims to synthesize, characterize, and then analyze its theoretical properties. On the other hand, the biological activity of the considered complex was evaluated to determine whether or not it possessed antibacterial activity.

## 2. Results and Discussion 

### 2.1. Chemistry 

**Scheme 1:**
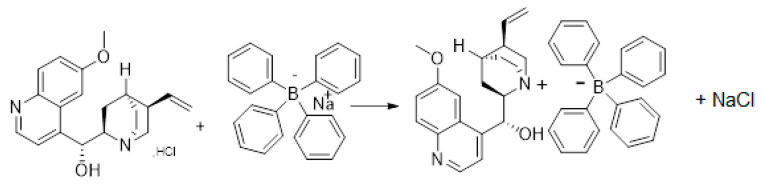
The synthetic pathway of the tetraphenylborate salt of quinine.

The title compound, (R)-(6-Methoxyquinolin-4-yl)[(1S,2S,4S,5R)-5-vinylquinuclidin-2-yl]methanol-tetraphenylborate complex, was produced in 81% of the reactions between sodium tetraphenylborate and (R)-(6-Methoxyquinolin-4-yl)[(1S,2S,4S,5R)-5-vinylquinuclidin-2-yl]methanol hydrochloride (quinine HCl) in deionized water at room temperature according to the following (Scheme 1). 

**Figure 1:**
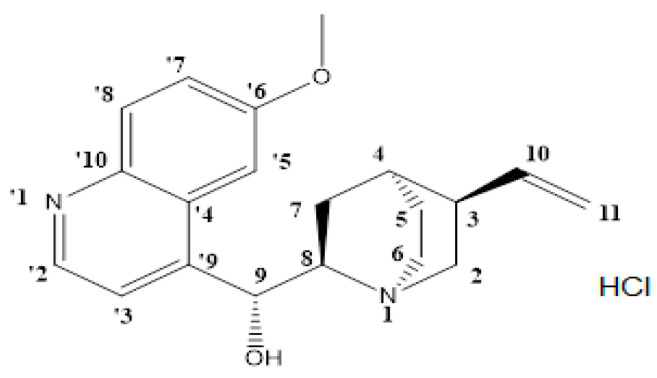
Chemical structure of quinine HCl.

Based on the chemical shifts and integrals, several resonances in the ^1^H-NMR spectrum of the quinine-tetraphenyl borate complex can be positively or tentatively identified (Figure 1). The methoxy proton was found as a singlet signal at 3.9 ppm. This spectrum displays the 14 protons that make up the quinine molecule’s vinyl quinuclidine moiety. Vinyl protons are present at 5.02–5.42 ppm, whereas quinuclidine protons are responsible for the resonance peak at 1.48–2.07 ppm as well as 2.7–3.6 ppm. The aromatic protons of tetraphenyl phenyl occurred between 6.80 and 7.38 ppm, while the quinolone proton spanned from 7.50 to 9.96 ppm. In the ^13^C NMR spectrum of the target complex, the OCH_3_ group is represented by a peak at 56.63 ppm, while the quinuclidine resonance peaks are located at 18.35, 24.36, 27.18, 52.97, 59.59, and 66.92 ppm, and the vinyl moiety resonance peaks are located at 102.24 and 132.03 ppm. Between 117.20 and 164.49 ppm, the aromatic carbons of quinoline and tetraphenyl rings were found. The ESI-MS (+ve) of the investigated compound revealed a peak at *m/z* 325.19 equal to (quinine^+^) in addition to the peak at *m/z* in addition to the peak 319 (Ph)_4_B^−^. The spectra of the titled ion-pair compound are inserted in Section 2.3.3, which shows maximum absorption at 310, 260, and 220 nm. 

### 2.2. Antimicrobial Activities

Table 1 displays the results. The disc diffusion test findings showed antibacterial activity against gram-positive bacteria (*Staphylococcus* ATCC 25923 and *Bacillus subtilis* ATCC 10400) and yeast (*C. albicans* ATCC 90028). The suggested compound had very poor antibacterial activity against gram-negative bacteria (*E. coli* ATCC 25922 and *Pseudomonas aeruginosa* ATCC 27853). The MIC was tested on the sensitive ATCC strains (*S. aureus* ATCC 25923, *B. subtilis* ATCC 10400, and *C. albicans* ATCC 90028). The proposed compound has the highest MIC against the sensitive ATCC strains. The MIC values for *S. aureus* ATCC 25923, *B. subtilis* ATCC 10400, and *C. albicans* ATCC 90028 are 8 μg/mL, 16 μg/mL, and 16 μg/mL, respectively.

### 2.3. Computational Study

#### 2.3.1. DFT Optimization

**Figure 2:**
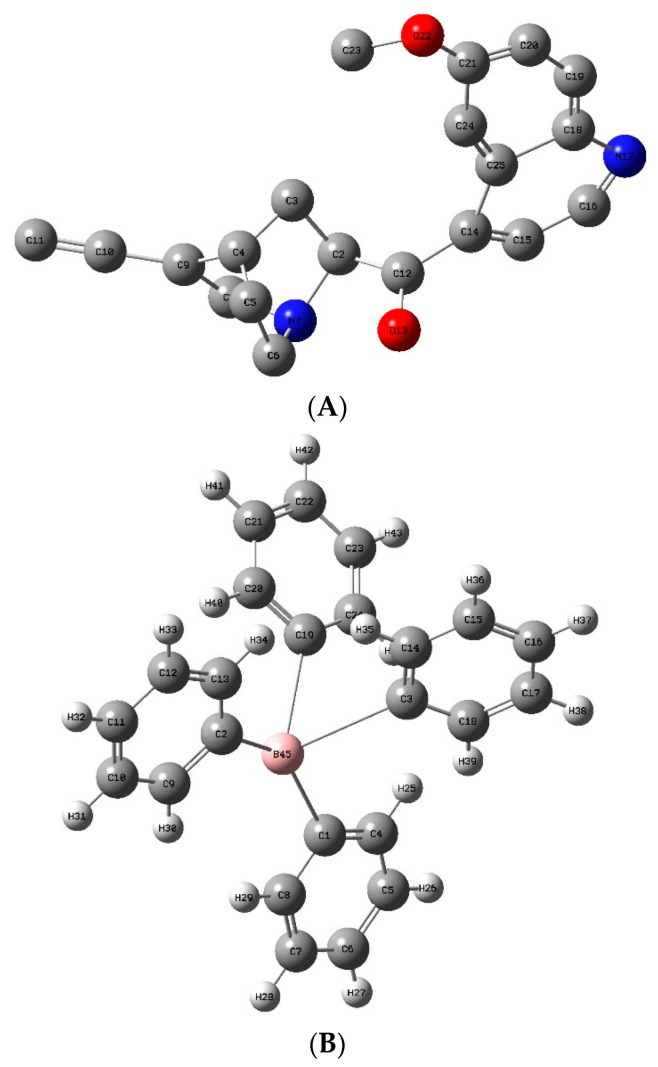

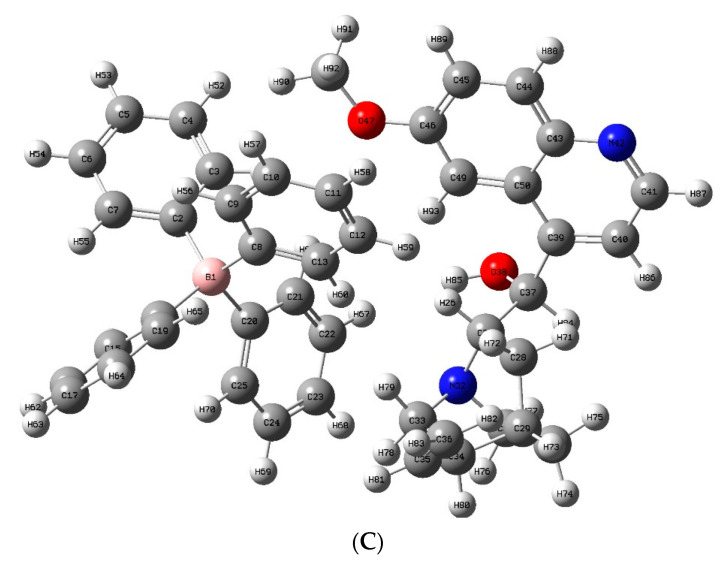
B3LYP/6–311G(d,p) optimized geometries of the molecules: (**A**) quinine cation (**B**) tetraphenylborate anion, (**C**) quinine cation, and tetraphenylborate anion.

The interaction of two molecules, quinine cation, and tetraphenylborate anion, to produce the complex was examined at 298 K using B3LYP under base 6-311++G(d,p). The computations were performed in a vacuum. Figure 2, shows the geometries that best characterize energetically ideal ion-pair combinations, their thermodynamic stability, and their bond interactions. The ideal cation structure of quinine can be seen in Figure 2A, while the ideal anion structure of tetraphenylborate can be seen in Figure 2B. The salt-optimized geometry can also be seen in Figure 2C. According to the optimized structures of the complexes obtained in Figure 2, boron (B^−^) and nitrogen in piperidine (N^+^) of the two molecules tetraphenylborate and quinine, respectively, are close to each other, while other rings in quinine and the two aromatic rings of tetraphenylborate are parallel.

#### 2.3.2. Interaction Energies (IEs)

For determining the most stable configurations and binding energies of structures, the B3LYP level with the 6–311G(d,p) basis set is frequently used. The complexation energy of the Q-TPB complex in the gas phase was, corrected and BSSE energy of −124.72 kcal/mole, −119.85 kcal/mole, and 0.00834. Accordingly, the complexation energy reveals low formation energies, indicating that both complexes are stable. Furthermore, the complexation energy of the complex is negative, showing that it can be produced spontaneously and that the results are consistent with the experimental procedure.

#### 2.3.3. Electronic Transitions by UV/Visible

**Figure 3:**
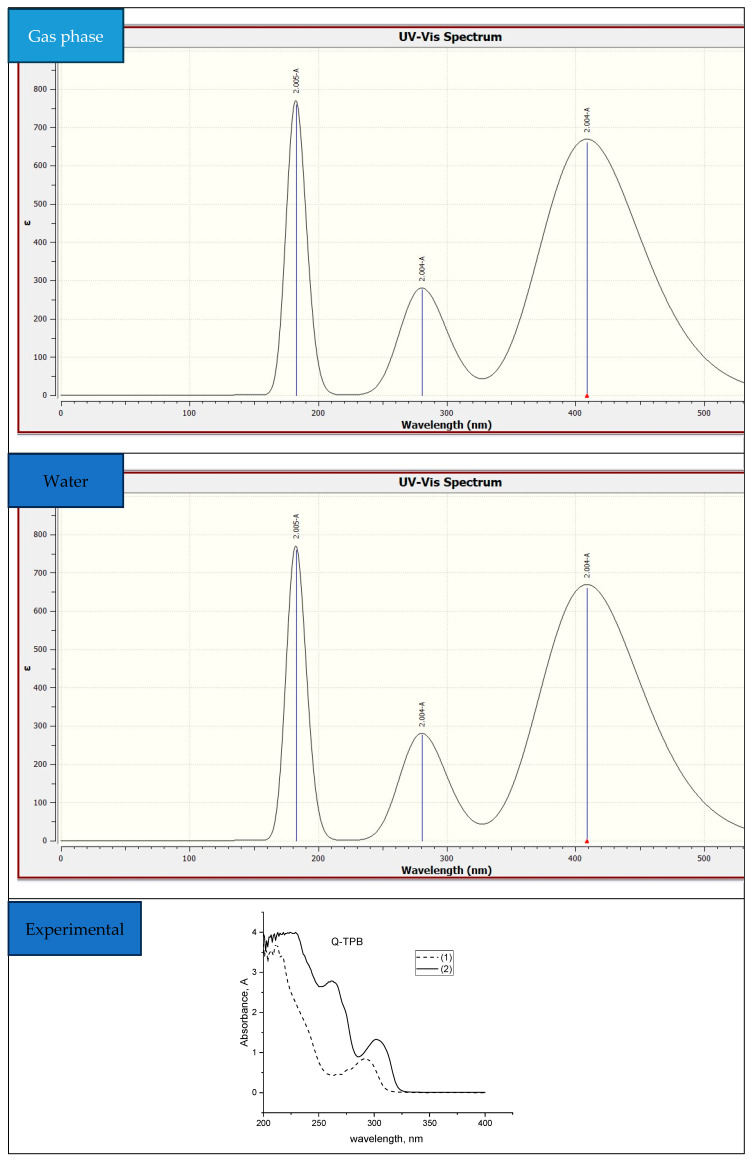
TD-DFT calculated absorption spectra in the gas phase and water solvent and experimental absorption spectrum of the title complex in water solvents at room temperature.

The time-dependent density functional theory TD-DFT is a method widely used to determine the absorption wavelengths which corresponds to vertical electronic transitions in a relatively short time from the optimized ground state structure [29,30]. The electronic spectra analysis of the aforementioned structure was conducted in both the gas and water solvent phases using the time-dependent-DFT (TD-DFT) IEFPCM model at the B3LYP/6–311G(d,p) level theory on the optimized ground state geometry. Nonetheless, the acquired absorption spectra were investigated at room temperature in aqueous solvents with a 50 ppm concentration, and the resulting plots are presented in Figure 3. The absorption spectrum depends on the nature of the interaction between quinine and tetraphenyl borate.

Figure 3 shows that the electronic transitions between the HOMO orbital and the empty LUMO orbitals correspond to these absorption bands at different energy levels. The three absorption bands observed in the Q-TPB complex correspond to the following electronic transitions: between H → L, H → L + 1, and H-1 → L with excitation energies of 3.03, 4.42, and 6.79 eV, which correspond with the maximum wavelengths of 408.9, 280.6, and 182.5 nm, respectively. The different orbitals corresponding to the observed transitions are the HOMO orbitals, which are represented by the delocalized π orbitals of the TPB molecule. The L and L + 1 orbitals are located on the vacant π* orbitals of the quinine. These excitations correspond to intermolecular π → π* transitions, which reflect the ionic interaction between TPB and quinine.

**Figure 4:**
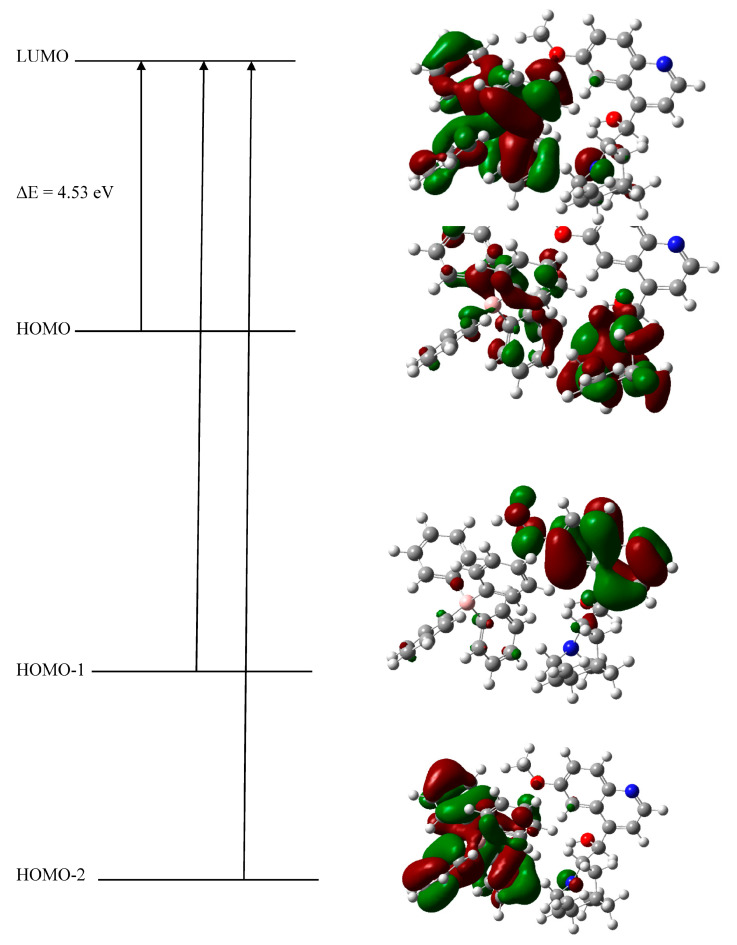
Electronic absorption in the gas phase is facilitated by band gaps (ΔE) and frontier molecular orbitals in Q-TPB complex.

The dipole moment (μ,D), main absorption energy (E), oscillator strength (ƒ), maximum wavelength (λ_max_), electronic transition of excitation energy, and atomic orbital contribution were calculated in the solvent and gas phases at room temperature. The corresponding data are shown in Table 2. According to Table 3, the energies of complexation in a vacuum and in water are negative, thus indicating that the complex formed is stable with an energetic favor in water of 1.1 kcal/mol. This preference is mainly due to the increase in the polarity of the medium (μ in water = 6.96 D), which favors the establishment of many electrostatic interactions between the two molecules TPB and quinine. The energy difference Δ(EHOMO − ELUMO) between the energies of the frontier orbitals is a direct measure of the stability of a molecule or a complex (Figure 4). The greater the absolute difference in value, the more stable the molecule or complex is and resists light radiation. In our study, the energy difference Δ(E_HOMO_ − E_LUMO_) of the Q-TPB complex studied in water is greater than that in a vacuum, confirming the stability of the Q-TPB complex in water over a vacuum. The dipole moment is a determining factor of the polarity of a molecule. In our case, the polarity of the complex increases with the polarity of the medium, it is 6.95 D in water and 2.45 D in a vacuum.

#### 2.3.4. Vibrational Frequencies in IR-Spectrum

Table 4 displays the selected typical vibrational band values along with the measured values. The calculated OH group stretching frequency appears at 3552 cm^−1^ in the infrared spectrum of complexes, while it is measured experimentally at 3487 cm^−1^. The calculated and measured vibrational values agree. The table below summarizes (Table 4) the evaluation of the peaks peculiar to each final structure’s spectrum as revealed by actual and hypothetical spectral IR data, as well as a beneficial correlation discovered within. 

#### 2.3.5. ^1^H NMR Chemical Shifts

A comparison of the calculated ^1^H NMR spectra of the complex with both configurations was performed based on B3LYP/6–311+G(d,p) in DMSO which is presented in Table 5. There is a very close match between the observed and computed chemical shifts. Based on the van der Waals interaction with TPB phenyl rings, a calculated deviation was observed. 

#### 2.3.6. Mulliken Charge

Another preliminary means attesting to the formation of a complex from two free reactants is the comparison of the corresponding Mulliken charges of the different atoms. Table 6 brings together the results, which show the modifications to the charges. The atoms most affected by Mulliken charges are B1, N32, O38, N42, O47, C48, C49, and C50. In the TPB molecule, B1 has a charge of −0.664 u.a., and quinine has the following charges: 0.228 u.a., −0.398 u.a., −0.538 u.a., −0.539 u.a., 0.316 u.a., −0.047 u.a., and 0.0.39 u.a.; while in the complex, these values become 0.204 u.a., −0.059 u.a., −0.095 u.a., −0.032 u.a., −0.286 u.a., 0.154 u.a., −0.265 u.a., 0.262 u.a., respectively. In the quinine molecule, the most positive atom N32 (0.228 a.u.) represents the site privileged to undergo a nucleophilic attack while in TPB molecule B1 is the atom most negative (−0.228 a.u.) is preferred to undergo an electrophilic attack, which is suitable for the formation of ionic interactions.

#### 2.3.7. Molecular Electrostatic Potential (MEP)

**Figure 5:**
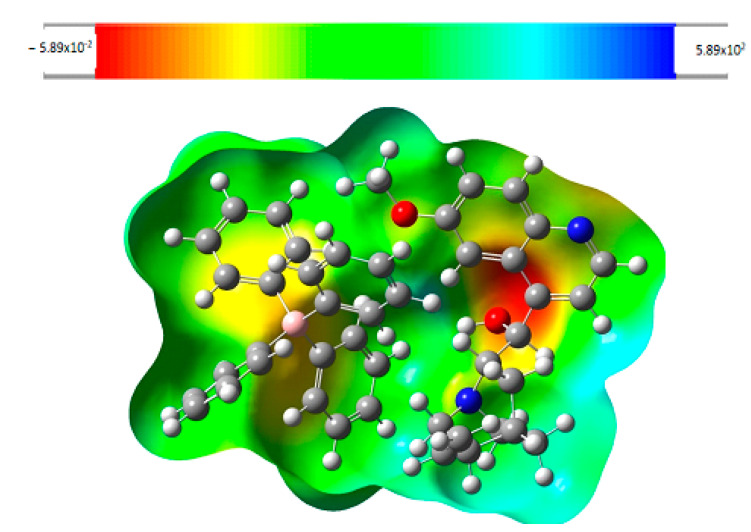
MEP of Q -TPB complex molecule.

Molecular electrostatic potential (MEP) analysis has been widely used as a tool for the understanding the effective localization of electron density in a molecule as well as to characterize the nature of the interactions and the existence of a charge transfer between the molecules. The different values of the electrostatic potential at the surface are represented by different colors. Increase in potential in the order red < orange < yellow < green < blue. The red represents the regions of the most negative electrostatic potential, which is related to electrophilic reactivity; blue represents regions of most positive electrostatic potential, which is related to reactivity nucleophile; and green represents the region of zero potential. The isosurface mapped by MEP for the Q-TPB complex is shown in Figure 5. As can be seen on the MEP map of the Q-TPB complex, the positive regions are located on the atoms of hydrogen, while the negative regions are located on the oxygen atoms. These sites give information about the region in which compounds can have intermolecular interactions.

## 3. Experimental

### 3.1. Materials and Instrument

The melting point was determined using a Gallenkamp melting point apparatus. NMR spectra were scanned in DMSO-*d*_6_ on a Brucker NMR (Brucker Inc., Phillandan, Switzerland) spectrometer operating at 500 MHz for ^1^H and 125 MHz for ^13^C. Chemical shifts are expressed in δ-values (ppm) relative to TMS as an internal standard. Coupling constants (J) are expressed in Hz. D_2_O was added to confirm the exchangeable protons. The mass spectrum was measured on an Agilent Triple Quadrupole 6410 QQQ LC/MS equipped with an ESI (electrospray ionization) source (Agilent Technologies, Inc., Santa Clara, CA, USA). A Shimadzu 1800 UV double beam spectrophotometer with quartz cell was used (Shimadzu, Kyoto, Japan), as was a PerkinElmer (PerkinElmer, Inc., Waltham, MA, USA), PE 2400 series II CHNS/O analyzer for elemental analysis.

### 3.2. Synthesis 

To a solution of (R)-(6-Methoxyquinolin-4-yl)[(1S,2S,4S,5R)-5-vinylquinuclidin-2-yl]methanol-tetraphenyl borate (quinine hydrochloride) (0.3969 g, 1 mmol) in deionized water (25 mL), a solution of sodium tetraphenylborate (0.3422 g, 1 mmol) in deionized water (25 mL) was added. The former white precipitate was filtered off, washed with cold deionized water, and dried over anhydrous CaCl_2_ white solid (yield > 81%). The melting point of the ion-associate complex was 177 °C, which is different than the reactants (160 °C and >310 °C) for quinine and sodium tetraphenyl borate, respectively. IR (KBr cm^−1^): 3300 cm^−1^ for OH. ^1^H-NMR (DMSO-d_6_) δ: 1.49 (1H, t, *J* = 10.0 Hz, H4), 1.86 (1H, dt, *J* = 10.0, 3.0, Hz, H5a), 2.56 (1H, sept., *J* = 1.5 Hz, H5b), 2.02–2.07 (2H, m, H7a,b), 2.72 (1H, q, *J* = 5 Hz, H3), 3.60–364 (2H, dt, *J* = 11.5, 3.5 Hz, H6a,b), 3.80 (2H, t, *J* = 9 Hz, H2), 3.9 (3H, s, OCH_3_), 5.02 (1H, d, *J* = 7.5 Hz, H11), 5.14 (1H, *J* = 12.5 Hz, H11), 5.85 (1H, ddd, *J* = 12.5, 7, 5 Hz, H10), 6.46 (1H, dd, *J* = 6.5, 1.5 H9), 6.80–7.38 (ArH for tetraphenyl), 7.50 (1H, dd, *J* = 9, 2 Hz, H7′), 7.66 (1H, d, *J* = 4.0 Hz, H3′), 8.01 (1H, m, H5′), 8.78 (1H, dd, *J* = 9.5, 4.7 Hz, H8′), 9.96 (1H, dd, *J* = 4.0, 1.0 Hz, H2′) ppm. ^13^C NMR (DMSO-*d*_6_) δ: 18.35, 24.36, 27.18, 52.97, 56.43, 59.59, 66.92, 102.24, 117.20, 120.33, 121.99, 125.75, 132.08, 136.26, 143.89, 148.62, 157.85, 163.57, 164.49. Elemental analysis: (C_44_H_53_BN_2_O_2_); cal. C 81%, H 8.13%, and N 4.29% found C78.98%, H: 8.12%, and N 4.19%.

### 3.3. Antimicrobial Susceptibility Testing

The antimicrobial activity of the proposed compound was tested against two gram-positive bacteria (*Staphylococcus aureus* ATCC 25923 and *Bacillus subtilis* ATCC 10400), two gram-negative bacteria (*Escherichia coli* ATCC 25922 and *Pseudomonas aeruginosa* ATCC 27853), and one yeast fungal strain (*Candida albicans* ATCC 90028). The disc diffusion method and minimum inhibitory concentrations (MICs) were used to test antimicrobial susceptibility.

#### 3.3.1. Disk Diffusion Method

The disc diffusion procedures for aerobic bacteria and yeast were carried out in accordance with the CLSI recommendations [31,32]. Three to five overnight fresh culture colonies were suspended in three mL of sterile normal saline. In a vortex, the suspension was extensively combined. Swiping in three directions dispersed the suspension equally across the entire surface of a Mueller–Hinton agar (MHA) plate (MHA with 2% glucose and 0.5 g/mL methylene blue was used for yeast). Before applying the compound-containing discs, the inoculated plates were allowed to dry. With the help of sterile forceps, each disc containing 100 mg of a substance was put on the surface of the inoculation plates. The antibacterial and antifungal quality controls were ciprofloxacin disc and fluconazole disc, respectively. For 20 h, the plates were incubated aerobically at 37 °C. After the incubation period, the results are documented by measuring the zone of inhibition (mm) with a ruler. The disc diffusion procedure was performed in triplicate, and the mean value was determined.

#### 3.3.2. Minimum Inhibitory Concentration (MIC)

The MIC procedures for aerobic bacteria and yeast were carried out in accordance with EUCAST recommendations [33,34]. The MIC for the active compounds was evaluated using the broth dilution method against standard ATCC sensitive bacteria. The working solution comprises 4096 g/mL DMSO. Twelve PJ sterile tubes were used. Each tube includes one mL of sterile Mueller–Hinton broth for bacteria and one mL of RPMI 1640 (with L-glutamine and a pH indicator but no bicarbonate) supplemented with glucose to a final concentration of 2% for yeast. The tubes were numbered one through twelve. In the first ten tubes, serial dilution was performed twice. Dilution was accomplished by transferring one mL of the component into the first tube and thoroughly mixing it. Then, one mL of the mixed solution from the first tube was pipetted into the second tube. This method was repeated until the tenth tube, and one mL of the tenth tube were discarded. One mL of the adjusted inoculum (5 × 10^5^ CFU/mL) was added to each of the numbered tubes. The 11th and 12th tubes were utilized as negative and positive controls, respectively. Ciprofloxacin and fluconazole were utilized as antibacterial and antifungal positive controls, respectively. The tubes were incubated at 37 °C for 20 h. The MIC results were manually recorded after the incubation time

### 3.4. Computational Details

Information is derived from computation. DFT has progressed from a rising star to a major player in computational quantum chemistry over the last several decades. DFT was used to calculate the conformational geometries of the target molecules. All computations were performed using the Gaussian 09 software package [35], and while efforts are being made to provide greater “general-purpose” capabilities, it is well known that particular characteristics are better suited to specific applications. Due to its advantageous combination of accuracy and computational cost, the B3LYP functional [36,37], which utilizes the basis set 6–311G(d,p) [38], has been successfully employed for reactivity research. Using the 6–311G(d,p) basis set, the initial geometry produced using typical geometrical parameters was decreased at the DFT level, with no limits on the potential energy surface [39]. To establish electronic absorption spectra (TD-DFT) on the optimized structure, time-dependent DFT was used [40].

## 4. Conclusions

In conclusion, the suggested ion-associate complex was easily produced by reacting quinine with sodium tetraphenyl borate in deionized water at room temperature. Different spectroscopic techniques, including UV, IR, NMR, and mass and elemental analyses, were used to characterize the complex’s structure, and their geometric structures were thoroughly investigated using experimental- and DFT-level theoretical calculations. The structure was characterized using spectroscopic methods (FT-IR and ^1^H-NMR). The electrostatic potential and geometric properties of the title complex were determined in the ground state using DFT/B3LYP/6–311G(d,p) basis sets. The energy differential (EHOMO-ELUMO) of the Q-TPB complex investigated in water is larger than that in a vacuum, indicating the Q-TPB complex’s stability in water over the vacuum. The dipole moment is a determining factor of a molecule’s polarity; in our case, the polarity of the complex increases with the polarity of the medium; it is 6.95 D in water and 2.45 D in vacuum. The complexation energy of the Q-TPB complex in the gas phase is −124.72 kcal/mole. As a result, the complexation energy for salt is low, indicating that the complex is stable. The estimated vibrational values and NMR data are in good agreement with the measured values. The antimicrobial study showed antibacterial activity against gram-positive bacteria and yeast. 

## Figures and Tables

**Table 1:** Inhibition zones and MIC values of chemical compounds against bacteria and yeast.

Compound	*S. aureus* ATCC 25923		*B. subtilis* ATCC 10400		*E. coli* ATCC 25922		*P. aeruginosa* ATCC 27853		*C. albicans* ATCC 90028	
	Inhibition Zone (mm)	MIC (μg/mL)	Inhibition Zone (mm)	MIC (μg/mL)	Inhibition Zone (mm)	MIC (μg/mL)	Inhibition Zone (mm)	MIC (μg/mL)	Inhibition Zone (mm)	MIC (μg/mL)
Q-TPB	25	16	20	32	9	ND	10	ND	20	32
Ciprofloxacin	34	0.25	30	≤0.25	32	≤0.25	29	0.25	ND	ND
Fluconazole	ND	ND	ND	ND	ND	ND	ND	ND	32	2

ND—not determined, and MIC—minimum inhibitory concentration.

**Table 2:** Energy of complexation (kcal/mol), dipole moment (μ,D), and energies of frontier orbitals calculated by the method B3LYP/6–311G++(d,p).

Parameters	Q-TPB	
Solvent	Gas Phase	Water
E_Total_	−45,833.05	−46,087.18
Dipole Moment	2.45	6.95
E_HOMO_	−6.12	−6.32
E_LUMO_	−1.99	−1.79
ΔE (E_LUMO_ − E_HOMO_)	4.13	4.53

**Table 3:** Maximum wavelength (λ max), oscillator strength (ƒ), assignment of electronic transition of excitation energy (HOMO(H) to LUMO(L)), atomic orbital contribution in gas and water phase.

Structure	Solvent	λ_max_	ƒ	Electronic Transition	Major % Contribution
Q-TPB	Gas phase	408.9 nm	0.162	H → L	97%
	Water		0.165	H → L	99%
	Gas phase	280.6 nm	0.077	H-2 → L H → L	84% 58%
	Water		0.069	H-2 → L H → L	85% 55%
	Gas phase	182.5 nm	0.186	H-1 → L H → L	97% 27%
	Water		0.190	H-1 → L H → L	97% 29%

**Table 4:** Experimental and calculated IR results, with determination of the correlation coefficient.

Band Assignments	Experimental	Calculated
νOH	3487 cm^−1^	3552 cm^−1^
νCH aliphatic	3055, 3000, 2984 cm^−1^	3034, 3015, 2990 cm^−1^
ν(CN)_py_	1622 cm^−1^	1621 cm^−1^
Phenyl	1509 cm^−1^	1510 cm^−1^
ν(OCH_3_)	1267 cm^−1^	1257 cm^−1^

Scaling factor: 0.972 for B3LYP/6–311G++(d,p).

**Table 5:** Experimental and theoretical (B3LYP/6–311+G(d,p)) GIAO ^1^H chemical shift values (with respect to TMS, all values in ppm) of the proposed complex.

Type	Label Numbers	Experimental Chemical Shift (ppm)	Calculated Chemical Shift (ppm)
ArH	71	1.48	0.92
ArH	73	1.49	1.43
ArH	74	1.51	1.48
ArH	75	1.86	1.61
ArH	72, 79	2.02	1.85
ArH	80	2.07	2.17
ArH, OH	78, 85	2.51	2.43
CH_3_	76, 92	2.72	2.98
CH_3_	91	3.35	3.20
CH_2_	77	3.60	3.38
CH	26	3.64	3.64
CH_3_	90	3.99	3.74
CH_3_	84	3.99	4.62
ArH	81	5.02	5.65
ArH	59	5.41	5.89
ArH	65	5.85	6.00
ArH	55	5.85	6.21
ArH	58, 64, 67	6.46	6.31
ArH	60	6.80	6.40
ArH	54, 52, 70, 57	6.80	6.51
ArH	53, 69	6.92	6.62
ArH	68	7.12	6.70
ArH	63, 89	7.12	6.78
ArH	86	7.19	6.87
ArH	93	7.19	6.94
ArH	62	7.38	7.04
ArH	56	7.38	7.11
ArH	66, 51	7.50	7.63
ArH	88	7.66	7.73
ArH	61	8.01	8.15
ArH	87	8.78	8.52

**Table 6:** Differences between Mulliken charges in quinine, TPB, and Q-TPB complex.

Atoms	Quinine	TPB	Q-TPB Complex
B1		−0.228	0.204
N32	0.228		−0.059
O38	−0.398		−0.095
N42	0.194		−0.032
O47	−0.539		−0.286
C48	0.087		0.154
C50	0.288		0.262

## Data Availability

All data involved in this study are included within the text.
